# Exploring unsafe sexual practices among truck drivers at Meerut District, India: a cross-sectional study

**DOI:** 10.4314/ahs.v21i2.9

**Published:** 2021-06

**Authors:** Ashish Pundhir, Arvind Shukla, Akhil Dhanesh Goel, Pooja Pundhir, Manoj Kumar Gupta, Pawan Parashar, Amit Mohan Varshney

**Affiliations:** 1 Department of Community Medicine Rama Medical College, Kanpur ,Uttar Pradesh India - 209217; 2 Department of Community Medicine and Family Medicine All India Institute of Medical Sciences-Raipur Raipur, Chattisgarh; 3 Department of Community Medicine and Family Medicine, All India Institute of Medical Sciences Jodhpur, Rajasthan, India; 4 Department of Internal Medicine Memorial Hermann Hospital Houston, Texas, USA; 5 Department of Community Medicine Subharti Medical College Meerut, Uttar Pradesh, India; 6 Department of Community Medicine Maharani Laxmibai Medical College Jhansi, Uttar Pradesh

**Keywords:** Unsafe sex, truck drivers, psychoactive substance, HIV, AIDS

## Abstract

**Background:**

Despite implementation of HIV prevention programmes for truck drivers in India, unsafe sex behavior among truck drivers has been documented.

**Objective:**

The objective of this study was to assess knowledge about HIV Transmission and modes of prevention, pattern of condom use with high risk partners and explore the practice of unsafe sex and its risk factors among truck drivers.

**Methods:**

This exploratory cross-sectional study design was conducted on a recruited convenient sample of 100 truck drivers above 18 years from March to May 2015. Binary logistic regression was used to compute unadjusted odds ratio [95% Confidence Interval] for establishing association of risk factors with unsafe sex.

**Results:**

Overall, only 7% had complete knowledge about HIV/AIDS transmission and prevention. 54% of truck drivers have sex with a high risk partner (commercial sexual worker or men having sex with men) and thirty-eight percent reported unsafe sexual practices due to inconsistent condom use with them. The various risk factors found significantly associated with unsafe sex were mean age of first intercourse (OR= 0.92, 95% CI: 0.75 – 0.97), access to pornography (OR = 4.4, 95% CI: 1.8 – 10.7) and conuming psychoactive substance before sex (OR = 4.06, 95% CI: 1.09 – 15.02).

**Conclusion:**

Socio-demographic, occupational factors, pornography access and consuming psychoactive substances seems to influence the sexual behaviour of truckers.

## Introduction

With 36.9 million people living with Human Immuno-deficiency Virus globally and absence of a definitive cure, the disease continues to remain a daunting public health problem of modern times. While the major burden is in Africa^1^, the estimated number of people living with HIV in India is 0.2 million.^2^

The past decade has seen a sharp fall in the incidence of HIV/AIDS in India. However, transmission still occurs especially in high-risk groups like those associated with truck and transport industry.^3^ The truck industry comprising of around 6 million truck drivers and their assistants has a considerable contribution to the national economy (5% GDP).^4^,^5^

Truck drivers are known to be at risk of a sexually promiscuous lifestyle and higher susceptibility to HIV/STI infection.^6^ Globally, many studies have documented high prevalence of sexually transmitted infections in truck drivers: Chen et.al. in 2006 reported a spectrum of STI like chlamydia(10.6%), gonorrhea(8.1%), herpes(4.4%) and syphilis(0.7%) among Chinese truck drivers whereas prevalence of HIV among long distance truck drivers was 47%.^7^,^8^ Various factors were found to facilitate transmission of HIV ranging from inconsistent condom use especially with commercial sexual worker and drug use.^9^,^10^ In 2017, the National AIDS Control Organization reported the prevalence of HIV among long distance truck drivers as 0.86%.^11^

There have been many HIV prevention programs (like Health Highway programme, ‘Kavach’ and Operation Lighthouse) implemented for the purpose of distributing condoms and promotion, behavioral change communication, care and counseling of truck drivers with STIs in India. Nevertheless, inconsistent condom use is reported among truck drivers with high-risk partners in India.^12^–^14^ To investigate the possible gaps in the knowledge about HIV transmission and prevention, patterns of condom use and explore the practice of unsafe sex and its risk factors, we embarked on this study among truck drivers.

## Method

### Study Design and Setting

This exploratory cross-sectional study was conducted from August 2014 to May 2015 at trans-shipment area in Meerut district, India where about 500 trucks are available at any given point of time.

### Sampling procedures and Sample size

A convenient sample of 100 truck drivers who had a valid driver's license from the transshipment area who had given their valid informed consent whereas drivers below the age of 18 years or not giving informed consent were excluded from the study.

### Study Tool and Procedure of Data Collection

The truck drivers were briefed about the purpose of the study, importance of their participation, its consequential benefit to other truck drivers and assured confidentiality. Regardless of the participation in the study, the truck drivers were given condoms and health promotion messages related to transmission and prevention of STI/HIV/AIDS. Interviews were conducted regarding unsafe sexual practices and misconception regarding condom use if any were explored. The interview took approximately 45 minutes to one hour.

The participant were interviewed using a pre-tested validated questionnaire for documenting socio-demographic-occupational profile, consumption of psychoactive substances, pornography access, high risk sexual partners [like commercial sexual worker(CSW) and men having sex with men(MSM)], intercourse type[vaginal, anal or oral sex], patterns of condom use, misconceptions about condom use and knowledge of transmission and prevention of STI/HIV/AIDs.

### Measures

Unsafe sex was defined as inconsistent use of condom with high-risk sexual partners like CSW and MSM irrespective of type intercourse-vaginal, anal or oral (fellatio) and practicing either cunnilingus or analingus or both.^15^ The inconsistent use of condom with high risk partners were assessed using the questionnaire.

Sex workers were those people who engage in occupational sexual behavior in exchange for economic rewards or other extrinsic considerations.^16^ Sex workers include male CSW, female CSW and transgender CSW. Men having sex with Men (MSM) were defined as men who have only sex with men. Participants who had sexual transactions with CSW within the last year were considered recent visitors to CSW. In India sero-prevalence of HIV infection in Female CSW, MSM and Transgender reported to be 1.56%, 2.67% and 3.14% respectively.^17^

Knowledge and misconceptions of truck drivers regarding modes of transmission and prevention were measured by using the pre-tested questionnaire. Knowledge of modes for transmission were considered to be complete if truck drivers responded with ‘yes’ to all of the following items (a) Transmission through sexual route (b) Transmission through blood transfusion (c) Transmission through needle sharing. If participants responded ‘yes’ to one or more but not all, they were considered to have incomplete knowledge of transmission. Similarly, knowledge of modes for prevention of HIV/AIDS were considered to be complete if truck drivers responded with ‘yes’ to all of the following items (a) Consistent use of condom (b) Prevented by having sexual relationship with one faithful uninfected sexual partner. Participants were considered to have no misconception about HIV/AIDS transmission, if they responded ‘no’ to all of the following items – HIV/AIDS is transmitted through (a) sharing the same meal (b) mosquito bite (c) sharing the same tools of infected person (d) sharing the same clothes with infected person (e) sharing the same toilet facilities with that of an infected person.

Truck drivers who answered yes to any of the following items were considered as having misconceptions about condom use - (a) Condoms can get lost in the women body/burst inside during sexual intercourse (b) Semen retention in condom can harm the man if it flows back into the penis (c) The use of condom increases the risk of HIV infection (d) Condoms are deliberately infected with HIV (e) Condom use reduces sensation and pleasure during sexual activity (f) Condom cause impotence/loss of erection and premature ejaculation (g) Condoms are small, tightly constricting and uncomfortable or too big that it will slip off (h) Condom use causes health related problem (i) Promotion of condom availability will increase promiscuity (j) Use of condom is against principles of religion. The ten aforementioned misconception addressing barriers to condom use was compiled from International Planned Parenthood Federation document (IPPF/UNFPA).^18^

Truck drivers occupational profile was detailed for factors like long distance travelling [≥400 kms], average number of days away from home, average resting time during the trip and age at the time of entry into this occupation(adult or adolescent entrant). They were enquired for consuming psychoactive substance like cannabis, alcohol, heroin, opium and poppy capsule before sex in past one year. The pattern of consumption was divided into 4 categories- Always, Mostly, Sometimes and Never. Similarly, they were asked if they had access toporn i.e. sexually explicit material in magazines, video, compact disc, mobile phone and/or internet. Truck drivers were asked if they had attended any health education session on HIV/AIDs organized by any Government or non-governmental organization.

Institutional ethical committee clearance was obtained before the start of the study.

### Data analysis

All the data were entered and analyzed using IBM SPSS statistics version 23 (Armonk,NY). Descriptive analysis was done to compute proportions for categorical data and mean and standard deviation for continuous data. Binary logistic regression (95% CI) was used for assessing correlates of unsafe sex, recent visit to CSW, inconsistent condom use with high risk-partners and correlates of anal and oral sex. A p<0.05 was considered significant.

### Ethical clearance

This study was a component of a dissertation and Subharti Medical College (Meerut,India) Institutional Committee approved the dissertation(SMC/PG-13/2013).

## Results

The mean age of the 100 participants was 33.7±8.8 yrs and 90% were literate. Of the 71 married truck drivers, 51(69.9%) reported premarital sexual partners and 15(20.5%) reported extramarital sexual partners.

Almost all of them had heard about HIV/AIDS and 40% of them were exposed to some HIV education programme. 76% had misconception about its transmission. Many felt it was transmitted through mosquito bite(61%) or sharing clothes(38%), toiletries(36%) and meals(31%) with HIV infected patients. Overall, 93% had incomplete knowledge about HIV/AIDS transmission. [Table 1]

**Table 1:** Socio-demographic profile and knowledge about HIV transmission and Prevention Mode (N = 100)

**Socio-demographic Profile**		
	Mean age	33.7 ± 8.8
	Married	72%
	Literate	90%
**Heard about AIDS and Exposure to Intervention Programme**		
	Heard about AIDs/STD	98%
	Exposed to HIV/AIDs/STD Intervention Programme	40%
**Knowledge about HIV Transmission and Prevention mode**		
	Knowledge about transmission through sexual route	95%
	Knowledge about transmission through blood transfusion	92%
	Knowledge about transmission through needle sharing	96%
	Knowledge about prevention through consistent condom use	95%
	Knowledge about Prevention by having one faithful partner	98%
**Misconception about HIV Transmission**		
	Sharing the same meal	31%
	Mosquito bite	62%
	Sharing the same tool of infected person	23%
	Living in the same room with infected person	24%
	Sharing the same clothes with infected person	38%
	Shaking hands with infected person	23%
	Using the same toilet facilities that of infected person	36%
	Misconception about HIV transmission	76%
**Incomplete knowledge about Transmission and Prevention of HIV/AIDs**		93%
**Incomplete Knowledge about Transmission and Prevention of HIV/AIDS :** Any participant with neither complete knowledge about HIV transmission mode nor complete knowledge about prevention mode and having misconception about HIV transmission mode.		

**Figure 1:**
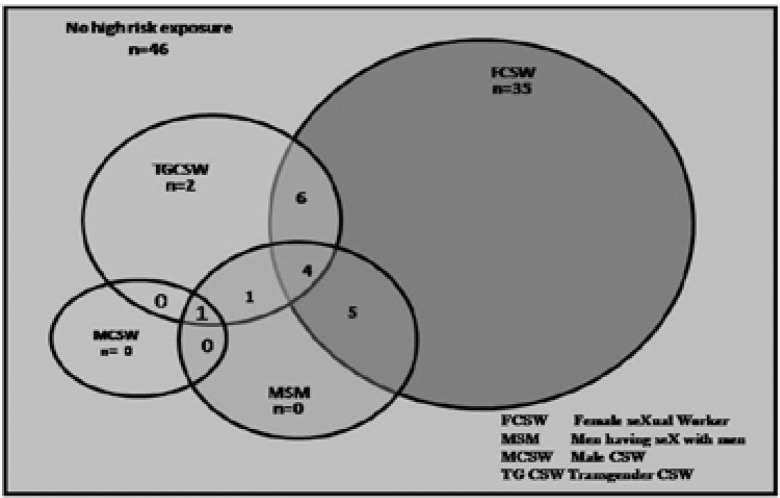
Patterns of Sexual encounters of truck drivers

Of 100 participants, 54 had sex with high-risk partners. Most of them had sex with female CSW (n=35) followed by transgender CSW (n=2) . Seventeen participants had multiple high risk sexual partners (n=6) with transgender CSW and Female CSW(n=5) with female CSW and MSM, 4 with transgender CSW, female CSW and MSM, two with transgender CSW and one each with MSM and transgender CSW and Male CSW, MSM and transgender CSW. [Figure 1]

**Figure 2:**
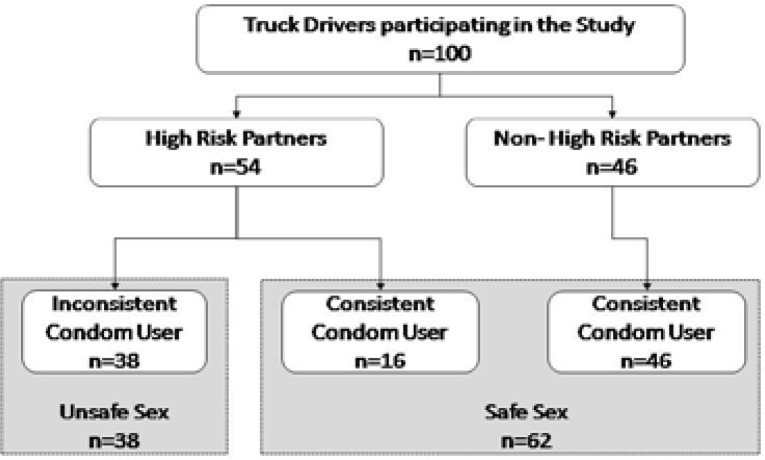
Profile of Study Participants

38 truck drivers practiced unsafe sex behaviour as they inconsistently used condom with high risk partners. Of 62 partners practicing safe sex, 16 consistently used condom with high risk partners. [Figure 2]. Unsafe sex was significantly associated with earlier age at first intercourse (p=0.017). Porn access (OR = 4.43, 95% CI: 1.84–10.74) and consumption of psychoactive substance before sex (OR = 4.06, 95% CI: 1.09–15.02) had statistically significant association with unsafe sex. [Table 2]

**Table 2:** Risk factors of unsafe sex

	Unsafe sex [N = 38]	Safe sex [N = 62]	OR (95% CI)	P-value
**Age, Mean ± SD**	35.9 ± 10.6	32.5 ± 7.3	1.046(0.997–1.097)	0.06
**Age of First Intercourse, Mean ± SD**	17.68 ± 3.65	19.63 ± 3.71	0.85 (0.75–0.97)	0.017
**Married**	28 (73.7%)	45 (72.6%)	1.06 (0.43–2.63)	0.904
**Literate**	35 (92.1%)	55 (88.7%)	1.49 (0.36–6.13	0.585
**Adolescent Entrant**	11 (28.9%)	20 (32.3%)	0.86 (0.36–2.06)	0.728
**Travelled Long distances**	30 (78.9%)	47 (75.8%)	1.12 (0.45–3.17)	0.717
**Average time away from home more than** **10 days**	12 (31.6%)	17 (27.4%)	1.22 (0.51–2.95)	0.657
**Resting time during journey more than 10** **hours**	16 (42.1%)	19 (30.6%)	1.64 (0.71–3.81)	0.245
**Porn access**	28 (73.7%)	24 (38.7%)	4.43 (1.84–10.74)	0.001
**Consumes Psychoactive substance before** **sex**	35 (92.1%)	46 (74.2%)	4.06 (1.09–15.02)	0.036
**Frequency of any psychoactive substance** **use before sex**				
Mostly to always consumes Psychoactive substance before sex	10 (33.3%)	2 (5.9%)	8.00 (1.5–40.0)	0.012
Never to sometimes consumes Psychoactive substance before sex	20[66.7%)	32(94.1%)	Reference	
**Consumes Alcohol before sex**	24 (63.2%)	13 (21.0%)	6.46 (2.63–15.89)	<0.001
**Consumes Cannabis and other** **psychoactive substance (Opium,Poppy,** **Capsule, Marijuana, Heroin)**	12 (31.6%)	11 (17.7%)	2.14 (0.83–5.50)	0.114
**Consumes Cannabis and other**	5 (13.2%)	5 (8.1%)	1.73 (0.47–6.41)	0.414
**psychoactive substance (Opium,Poppy,**	12 (31.6%)	11 (17.7%)	2.14 (0.83–5.50)	0.114
**Capsule, Marijuana, Heroin)**				
**Consumes Cannabis and other**				
**Psychoactive substance before sex**	5 (13.2%)	5 (8.1%)	1.73 (0.47–6.41)	0.414
**(Marijuana, Bhang,Opium,Poppy Capsule)**				
**Smokes and Chews Tobacco**	29 (76.3%)	46 (74.2%)	1.12 (0.44–2.86)	0.812
**Misconception about condom use**	28 (73.7%)	38 (61.3%)	1.76 (0.73–4.28)	0.207
**Exposure to**				
**Education Programmes related to**	17(44.7%)	23(37.1%)	1.37(0.604–3.12)	0.450
**HIV/AIDS**				
**Incomplete Knowledge about HIV** **Transmission and Prevention Mode**	33 (91.7%)	58 (93.5%)	0.46 (0.11–1.81)	0.264
**Not always carrying condom during travel**	27 (71.1%)	35 (63.6%)	1.40 (0.58–3.42)	0.457

The majority consumed a psychoactive substance before sex(92.1%) and unsafe sex was higher among those consuming a psychoactive substance always and mostly before sex (OR=8.0, 95%CI: 1.5–40). Two-thirds consumed alcohol before sex which was significantly associated with unsafe sex (OR=6.46, 95%CI: 2.63–15.89). [Table 2]

Of 38 participants practicing unsafe sex, 73.7% had some misconception about condom use – 21 felt it reduces pleasure: “*There is no pleasure and since seminal fluid remains confined to condom*.”, four felt it causes sexual dysfunction: “*Condom is a barrier, it's like you need a wire to light a bulb, likewise when there is direct contact of penis with vagina then penis is erected*.”, three felt its use promotes promiscuity: “*Condom affects the sexual satisfaction of both partners, they will keep on engaging with different partners till their sexual appetite is met*.” One each felt semen retention causes retrograde flow in the penis: “*On account of pressure, semen can retrogradely flow back into penis causing papules*”, two felt it causes health related problems: one felt it increased risk of HIV infection: “*Yes because they get ruptured*.”

### Pattern of Condom use with High risk partners

Of 54 participants having sex with high risk partners, 38 inconsistently used condoms. This was especially higher in the 11 MSM of whom 9 (81.8%) reported inconsistent condom use during fellatio and 8 (72.7%) during anal sex. With CSW, inconsistent condom use was highest during fellatio (84.6%) followed by anal sex (42.9%) and (38.8%).

### Risk factors for Anal and Oral sex

Access to porn (OR=4.24, 95%CI: 1.6–11.5) and consumption of psychoactive substance before sex (OR=9.35, 95%CI: 1.2–73.8) was significantly associated with practice of anal sex. Similarly, having access to porn(OR = 5.48, 95% CI: 2.27–13.26) and consuming psychoactive substances before sex (OR = 4.04, 95% CI: 1.23–13.22) was significantly associated with oral sex. [Table 3]

**Table 3:** Risk factors for anal and oral sex

	Anal sex (N=28)	No Anal sex (N=72)	OR 95% CI	p-value	Oral sex [N =42]	No Oral sex [N =56]	OR (95% CI)	p value
**Mean Age** **(SD)**	33.5 ±8.7	34.2±8.9	1(0.955–1.058)	0.848	34.6±8.1	33.5±9.4	0.985(0.941–1.031)	0.518
**Married**	25(88.3%)	48(68.6%)	3.83(1–14)	0.043	32 (76.2%)	40(71.4%)	1.28(0.512–3.2)	0.598
**literate**	27(96.4%)	61(87.1%)	3.74(0.481–33.02)	0.206	39(92.9%)	49(87.5%)	1.85(0.451–7.65)	0.392
**Porn access**	21(75%)	29(41.4%)	4.24(1.59–11.5)	0.004	31(73.8%)	19(33.9%)	5.48(2.27–13.26)	<0.001
**Consumed** **psychoactive** **substance** **before sex**	27(96.4%)	52(74.3%)	9.35(1.18–73.8)	0.011	26(83.9%)	18(56.2%)	4.04(1.23–13.22)	0.021

Of 28 truck drivers practicing anal sex, majority were with CSW (50%) followed with men (39.3%) and wife (21.4%). Forty two reported practice of oral sex and of which 90.5% practiced fellatio mostly with CSW (68.4%) followed by premarital partners (23.7%), MSM (23.7%). Four (10.5%) had consistently used condoms during the act of fellatio and reveals 23.8% (10) had practiced cunnilingus, with most of them with their wives (70%) following with premarital (30%) and extramarital (20%). Only 2 drivers reported practicing analingus with their wives.

### Details of sexual relationship with CSW

The mean age of truck drivers having intercourse with CSW was 20.8(4.8). Of 98 truck drivers, 54(55.1%) had sexual transaction (including oral and anal Sex) with CSW. Twenty Two (40.7%) drivers visited CSW within past one year (recent visitor). The majority (54.5%) visited one to five times and 22.7% reported visiting more than 15 times.

Co-workers (36.4%), friends/relatives (9.1%) and other sources (63.6%) being roadside restaurant (‘Dhaba’) owners, flashing of torch lights during journey were a source of information to truck drivers having recent sexual encounters with CSW. Mostly, sexual transaction with CSW were during journey (54.5%), loading and unloading of trucks (13.6%) and 9.1% near roadside restaurant (‘Dhaba’).

The odds of recent visit to CSW were higher in drivers remaining away from home for more than 10 days (OR=4.3, 95%CI: 1.28–14.67) and was statistically significant. [Table 4]

**Table 4:** Risk factors for Recent visit to CSW

	Recent visit to CSW		OR (95%CI)	P - value
	Yes	No		
	(N =22)	(N = 32)		
**Mean age of First Intercourse with** **CSW**	21.4±4.64	20.5±4.89	1.045[0.930–1.173]	0.459
**Literate**	21(95.5%)	27(84.4%)	3.89(0.422–35.9)	0.231
**Unmarried**	7(31.8%)	8(25%)	1.4(0.421–4.66)	0.583
**Adolescent entrant**	(40.9%)	(28.1%)	1.76(0.562–5.572)	0.330
**Ever travelled Long distance**	20(90.9%)	22(68.75%)	4.54(0.887–23.3)	0.069
**Average number of days away from** **home when on trip more than 10** **days**	11(50%)	6(18.8%)	4.33(1.28–14.67)	0.018
**Approximate resting time during** **journey more than 10 hrs**	7(33.4%)	12(37.5%)	.833(0.262–2.647)	0.757
**Psychoactive substance**	22(100%)	26(81.2%)	-	0.031
**Exposure to HIV** **Intervention Programmes**	8(36.4%)	16(50%)	1.75(0.576 – 5.316)	0.324

The odds of inconsistent condom use is higher among truck drivers recently visiting CSW more than 5 per week as compared to those visiting less than 5 times a week (OR=11.6, 95%CI: 1.5–89.2,p<0.05). High risk behaviour of inconsistent condom use is higher among truck drivers (recent visitors) having frequent sexual transaction with CSW. Hence, through health education this issue is to be addressed.

### Pattern of Condom use with Truck drivers and Reasons for Refusal

Of 54 truck drivers, 22 (57.4%) used condom inconsistently with CSW. Of 22 inconsistent user of condom, 8(36.4%) truck drivers inconsistently used condoms because it was not available with them, 6(27.3%) felt condom reduces pleasure: “*They(CSW) had condoms but I paid them extra to do it without condom, so I can get more pleasure. Moreover, my peer advised me not to use condom*.” 4(18.2%) due to non-availability of condom with CSW, 2(9.1%) because the thought of using it did not occur and 2 lacked knowledge about HIV/AIDs: “*I had no awareness about condom*”.

## Discussion

Around two-fifth subjects practiced unsafe sex with more than half having sex with high risk partners. An earlier mean age of first intercourse, porn access, consumption of psychoactive substance were associated with practice of unsafe sex. Around three-fourths of them had misconception about condom use. Overall 7% had complete knowledge about transmission and prevention mode of HIV/AIDS.

This study documents more than half of the participants had history of life-time exposure to CSW. Other studies in India document life-time exposure varying from 40.1% to 58.6%.14,^19^–^21^ Studies from countries like Hong Kong, Namibia and South Africa have reported exposure rate to CSW as 17%–30%.^22^–^24^

Among both life-time exposure and recent visitors to CSWs, more than half of participants consistently used condoms. 72% of truckers in Siliguri-Guwahati Highway consistently used condoms with CSW.19 In the present study, more than half of the participants with recent sexual transaction with CSW approached them during journey which is consistent to findings from Namibia where most truckers interacted with CSWs while on roads and away from home.^24^

50% of adolescent participants recently had intercourse with CSWs compared to 36.1% adult entrants, indicating that adolescent entrant are more likely to engage with CSWs. This is consistent to Mishra et al. where adolescent entrants were more likely than adult entrants (42.6% vs. 27.2%) to have sex with CSW.^25^

In our study, 47.4% long distance drivers had sex with CSWs including gender other than female and 44.4% long distance drivers had consistently used condoms. A study done elsewhere in India among long distance truck drivers at seven trans-shipment locations along four routes revealed 24% having sex with female sexual workers and 75% consistently used condoms.^26^

The current study indicates practice of unsafe sex was higher in married truckers but recent intercourse with CSWs was higher in unmarried truckers. It also reported a significant association for illiteracy and staying away from home for more than 10 days with recent visit to CSW. This is similar to Pandey et al who indicated engagement with CSWs was higher in unmarried and illiterate truckers and those who stayed away from home for more than 10 days.^27^,^28^

Singh R and Joshi H observed that reasons for not using condoms with CSW were non-availability (37.5%), uneasy feeling during intercourse (35.7%), did not feel it necessary (14.3%) which is also similar to the findings in our study.^14^

Our study reports more than a quarter of participants practiced anal sex and more than half indulged with CSW. A study evaluating partner correlate specific of anal sex in Tamil Naidu reported 5% truck drivers practicing receptive or insertive anal intercourse with a male or transgender.^29^

The present study documents 42% of participants practiced oral sex and more than half indulged in fellatio with a CSW and about a quarter with MSM. In India, there seems to be no recent study on oral sex practices except for Manjunath et al (2002) who recruited truckers from STD clinic along the Pondicherry-Tindivanam Highway which reported 4.1% truckers practiced oral sex.^30^

Unsafe sex was higher in participants consuming psychoactive substances and alcohol before sex. Among truckers in Namakkal, Tamil Naidu 24% consumed alcohol before sex.^29^ Consumption of psychoactive substances among trucker have been documented in Brazil, USA, Azerbaijan, Guinea, Nigeria) and Hong Kong and shown to influence unsafe sexual practices.^23^,^25^,^28^,^31^.^32^ This study also documents consumption of psychoactive substances like opium, heroin before sex. In Uganda, findings indicate alcohol use before sex and the belief that condom use kills the mood for sex were key barriers to consistent condom use among truckers visiting CSW.^13^

A systematic review estimated the per act probability of acquiring HIV from infected partner from the following sexual exposure per 10000 exposure is 138 for receptive anal sex; 11 for insertive anal sex; 4 for insertive peno-vaginal sex; 8 for receptive peno-vaginal sex. However, transmission risk from both receptive and insertive oral sex was quite low.^33^

Unsafe sex was higher in participants who had misconception about condom use despite association being statistical insignificant and most of them believed it decreased pleasure. Less than one-tenth of our participants had complete knowledge about HIV transmission and prevention modes and 2 participants never heard about HIV/AIDS. A study among truck drivers in Tamil Naidu, India findings indicate 71% knew it could not be transmitted by bites of mosquitoes/insects whereas 76.8% knew it cannot be transmitted by sharing clothes and eating from the same utensil and overall 4% had correct knowledge of HIV/AIDS.29 Singh R. and Joshi HS.(2012) found 81.8% knew HIV can be transmitted through sexual route if condom not used, 66.9% through shared needles among drug users , 93.2% through contaminated blood transfusion and 86.1% through breast feeding. 49.3% had knowledge that it can be prevented by being faithful to regular partner. There were also misconception about route of HIV transmission - 21.3% thought it was transmitted through mosquito bite, 10.8% thought that it could transmit by living in same room, 9.8% thought by shaking hands and 2.7% sharing tools.14 Similary, misconception was documented in three-fourths of participants and nearly two-thirds believed HIV could be transmitted through mosquito bite and around one-fifth felt it was transmitted sharing same clothes, tools and shaking hands of a HIV infected patients.

The present study establishes the practice of unsafe sex was higher among those having access to porn, highrisk partners [MSM,CSW(male,female and transgender)] and inconsistent condom use with those visiting CSW often [more than five times per week] and elaborately describes partners in anal and oral sex as well as misconception about condom use. We assessed the association of socio-demographic details, porn access, misconception about condom use and consuming psychoactive substance use before sex with inconsistent condom use with high risky partners. Nevertheless, except for porn access the association was statistically insignificant. Overall, a cautious interpretation of association is warranted due to small sample size and convenient sampling procedure. Also, recall and social desirability bias cannot be completely ruled out.

## Conclusion

Younger age and occupational factors like type of study participant, average time away from home, pornography access and consuming psychoactive substances seems to influence the sexual behaviour of truckers. However, a rigourous emphasis on primary prevention should focus on new experience and adolescent entrants which can help in reducing unsafe sexual practices. Implementation science research should be undertaken to understand the barriers to preventive service offered from existing facilities provided by State AIDS Control Society and improve knowledge about HIV/AIDS transmission and prevention. A lasting impact on safe truck driver behaviour will require a multi-pronged behaviour change strategy targeting high risk subgroups like adolescent entrants and psychoactive drug abusers.
